# The IGF Hormonal Network in Endometrial Cancer: Functions, Regulation, and Targeting Approaches

**DOI:** 10.3389/fendo.2014.00076

**Published:** 2014-05-19

**Authors:** Ilan Bruchim, Rive Sarfstein, Haim Werner

**Affiliations:** ^1^Gynecologic Oncology Unit, Department of Obstetrics and Gynecology, Meir Medical Center, Kfar Sava, Israel; ^2^Department of Human Molecular Genetics and Biochemistry, Sackler Faculty of Medicine, Tel Aviv University, Tel Aviv, Israel

**Keywords:** insulin-like growth factors, IGF1, IGF1 receptor, endometrial cancer, uterine serous carcinoma, p53, BRCA1, biomarkers

## Abstract

Epidemiological as well as clinical and experimental data identified the insulin-like growth factors (IGF1, IGF2) as important players in gynecological cancers in general, and endometrial tumors in particular. The IGF1 receptor (IGF1R), which mediates the proliferative and anti-apoptotic activities of both ligands, emerged in recent years as a promising therapeutic target in oncology. However, most clinical trials conducted so far led to mixed results, emphasizing the need to identify biomarkers that can predict responsiveness to anti-IGF1R-targeted therapies. This article will review recent data regarding the role and expression of IGF system components in endometrial cancer. In addition, we will review data on the interplay between the IGF signaling pathway and tumor suppressors p53 and breast cancer susceptibility gene-1 (BRCA1). Anti-oncogenes p53 and BRCA1 play a key role in the etiology of gynecological cancers and, therefore, their interaction with IGF1R is of high relevance in translational terms. A better understanding of the complex mechanisms underlying the regulation of the IGF system will improve our ability to develop effective treatment modalities for endometrial tumors.

## Endometrial Cancer

Endometrial cancer is the most widespread gynecologic cancer in Western countries. Women have a 2–3% lifetime risk of developing this malignancy. More than 40,000 new cases were diagnosed in the USA in 2009 and more than 52,600 cases were estimated to be diagnosed in 2014 (1). As illustrated by these figures, the incidence of the disease has been increasing over the past decades, presumably because of the growing obesity epidemic. The impact of obesity and diabetes on endometrial cancer risk will be discussed below. Endometrial cancers are classified into two major categories based on histological parameters, clinical behavior and epidemiology (Table 1). Type I tumors are estrogen-related and account for more than 80% of the cases (2, 3). These tumors exhibit an endometrioid, well-differentiated morphology and are usually associated with a relatively good prognosis. Type II tumors, on the other hand, display a less differentiated phenotype and bear a worse prognosis. Uterine serous carcinoma (USC) constitutes the predominant histological class among Type II tumors. Although USC represents only 10% of all endometrial cancers, it accounts for more than 50% of all relapses. USC is considered as a high grade cancer and has a significantly poorer prognosis than endometrioid tumors, with a 5-year survival rate of 55% (4).

**Table 1:** **Classification of endometrial cancers**.

**TYPE I TUMORS**
80% of cases
Estrogen-dependent
Endometrioid, well-differentiated morphology
Usually good prognosis
Estrogen receptor positive, diploid, microsatellite unstable
Include KRAS and/or PTEN mutations
**TYPE II TUMORS**
10% of cases
Less differentiated phenotype
Often serous papillary histology
Worse prognosis
Often aneuploid with alterations in CDK2A, p53, and ERBB2

A number of molecular alterations have been linked to endometrial malignancies. Some of these defects are specific to either Type I or II tumors, although there is a certain overlap in some of the aberrations. Type I tumors are often estrogen receptor (ER) positive, diploid, microsatellite unstable and have KRAS, PTEN, and/or β-catenin mutations. Type II tumors are usually aneuploid, with alterations in CDK2A, p53, and ERBB2 (5, 6). Loss of ER and/or progesterone receptor (PR), loss of CDKN2A, and over-expression of p53 and ERBB2 are associated with decreased survival of Type II patients (7–10). Women with recurrent and/or metastatic endometrial cancer of either type have a poor prognosis, with a median survival of 7–12 months (11). These patients require more effective systemic therapy than is presently available. Currently, adjuvant and systemic treatment of recurrent and metastatic endometrial cancer are based on conventional chemotherapy and anti-hormonal treatment. As indicated above, the growing obesity epidemic in recent decades had a major impact on endometrial cancer incidence in most developed countries. The role of obesity, hyperinsulinemia, and diabetes on endometrial cancer biology will be described in the context of the insulin-like growth factor (IGF) system.

## IGF System in Normal Uterine Physiology

The IGFs constitute a network of cellular and secreted proteins with essential biological functions (12, 13). Since their discovery in 1957 by Salmon and Daughaday, the IGFs have attracted vast scientific awareness (14). Interest in this family of hormones, cell-surface receptors, and circulating and membrane-bound IGF-binding proteins (IGFBPs) stems mainly from the recognition that the IGF signaling pathways are involved in multiple, clinically relevant, pathophysiological processes in the areas of endocrinology, pediatrics, aging, and oncology (15–19). IGF1, which was initially identified by its ability to mediate the effects of growth hormone on cartilage sulfation and longitudinal growth, is produced mainly in the liver. Many organs, however, possess the biosynthetic machinery necessary to produce the hormone at various levels. Locally produced IGF1 and IGF2 are mainly involved in organ-specific, autocrine, and paracrine types of activities. Both IGF1 and IGF2 ligands activate a common receptor, the IGF1 receptor (IGF1R), which signals mitogenic, anti-apoptotic, and transforming activities. The IGF1R is a cell-surface tyrosine kinase receptor coupled to several intracellular secondary messenger pathways, including the *ras–raf* –MAPK and PI3K–AKT signaling cascades. The IGF1R is vital for cell survival, as illustrated by the lethal phenotype of mice in which the IGF1R gene is disrupted.

In the context of normal uterine physiology, the IGF axis has a major regulatory role. IGF1 has been identified as a potential mediator of the effects of estradiol on uterine growth and uterine IGF1 biosynthesis was shown to be directly regulated by local estradiol levels. Furthermore, IGF1-targeted gene deletion mice exhibit a disproportionate reduction in uterine size (16). It is unclear, however, whether this reduction is due to diminished estradiol production or, alternatively, to defective estrogen action due to lack of locally produced IGF1. Of importance, cyclic changes in IGF1 expression and signaling play key roles in regulating the transition of the premenopausal endometrium through the proliferative, secretory, and menstrual cycles. Finally, as described in the next section, there is evidence of a causative linkage between deregulated expression and activation of IGF system components and endometrial cancer.

## IGF System in Endometrial Cancer

Experimental, clinical, and epidemiological evidence indicates that the IGF signaling pathways are important mediators in the biochemical and molecular chain of events that lead from a phenotypically normal cell to one harboring neoplastic traits. While this general dogma applies to most human malignancies, the activity of the IGF hormonal network in gynecological tumors is strongly influenced by interactions with organ-specific factors, in particular steroid hormones. Studies by several groups have shown that IGF1 has a significant role in both Type I and II endometrial cancers, emphasizing the importance of altered IGF1R gene expression in the development of a malignant phenotype (20–23).

## Impact of Obesity and Diabetes on Endometrial Cancer

Given the structural and functional correlations between IGF1 and insulin, as well as the fact that both hormones employ identical signaling mediators, it is necessary to focus on the impact of obesity and diabetes on endometrial cancer. Diabetes mellitus type 2, a condition often associated with chronic endogenous insulin excess, is a well-established risk factor for certain types of cancer, including endometrial tumors (24). In addition, at least 40% of endometrial cancers can be attributed to excess body weight. Epidemiological studies have shown that the metabolic syndrome and its components (hyperlipidemia, obesity, and high blood pressure) constitute risk factors not only for diabetes and cardiovascular diseases, but for cancer as well (25). Women with a body mass index (BMI) above 32 kg/m^2^ have a relative risk of 4.0 for the development of endometrial cancer, and women with a BMI above 35 kg/m^2^ have a relative risk of 6.0, in comparison with lean women (BMI under 23). Given the fact that the conversion of androstenedione to estrone takes place in peripheral adipose tissue, obese women have elevated values of endogenous estrogen. High circulating estrogen levels may result in endometrial proliferation and hyperplasia leading, potentially, to enhanced cancer risk (24). However, elevated estrogen levels may not entirely account for the linkage between obesity and endometrial cancer, and other obesity-related factors were postulated to be involved in this connection. In particular, chronic hyperinsulinemia and insulin resistance were identified as major players in the link between obesity, lack of physical activity, development of ovarian androgen excess and, finally, endometrial cancer.

The insulin/IGF pathway, which is usually hyper activated in obesity and diabetes, is a major player in the chain of events linking the metabolic syndrome with cancer (12, 17, 26–28). The *insulin-cancer hypothesis* postulates that chronic hyperinsulinemia, a typical hallmark of diabetes, is one of the leading factors responsible for the obesity–cancer connection. In the context of endometrial cancer, high concentrations of circulating insulin can exert both direct and indirect effects that contribute to the development of the tumor. Directly, insulin promotes cell proliferation and survival through activation of the *ras–raf* –MAPK and PI3K–AKT pathways. Indirectly, insulin leads to changes in sex hormones, including increased estrogen levels, with ensuing reduction in IGFBP1 levels, a negative regulator of IGF1. The net result of IGFBP1 downregulation is a major increase in IGF1 activity.

## Circulating IGF1 Levels

Consistent with a key role in endometrial cancer initiation and development, Ayabe et al. (29) reported higher IGF1 and lower IGFBP1 levels in postmenopausal endometrial cancer patients. In contrast, Petridou et al. (30) reported that endometrial cancer was positively associated with IGF2 serum levels and inversely associated with IGF1. Another case–cohort study that included 250 incident endometrial cancer patients and 465 controls assessed the association between endometrial cancer risk and serum levels of IGF1, IGFBP3, insulin, and estradiol (31). Low levels of free IGF1 and high insulin levels were associated with endometrial cancer risk after adjustments for age, hormone therapy use, and estradiol levels. Both associations were stronger among obese patients, especially the linkage between insulin and endometrioid adenocarcinoma. However, other studies did not report correlations between endometrial cancer risk, IGF axis components, and insulin levels (32). In summary, the large degree of variability between studies reflects, on one hand, the complexity of this hormonal system and, on the other hand, the involvement of additional (hormonal or other) factors that can either positively or negatively impinge upon IGF axis components. An additional factor that may contribute to the conflicting reports is the difficulty to have a real estimation of IGF1 system activation through the dosage of IGF1 serum levels. A recently developed IGF1 kinase receptor activation assay (KIRA) allows assessing circulating IGF1 bioactivity by quantifying phosphorylation of tyrosine residues of the activated IGF1R after stimulation with human serum *in vitro* (33). In summary, more research is needed to firmly establish the diagnostic and prognostic value of circulating IGF1 in endometrial cancer.

## Tissue Expression of IGF System Components

A significant increase in IGF1R expression in biopsy specimens from hyperplastic endometrium and endometrial carcinoma, in comparison to proliferative endometrium, was reported by McCampbell et al. (21). This study included 10 women with proliferative phase endometrium, 7 women with secretory phase endometrium, and 17 postmenopausal women with endometrial complex atypical hyperplasia. Similarly, Hirano et al. (20) reported high IGF1R expression in all types of gynecological cancers. This study included specimens from 46 endometrial, 32 cervical and 20 ovarian cancers, and 28 normal endometrium. Elevated IGF1R mRNA expression was observed in 91.3% of endometrial cancers. The correlation between IGF1R and IGF2 expression levels with endometrial cancer stage was investigated in a study that included specimens from 59 endometrial adenocarcinoma cases, 10 endometrial hyperplasia cases, and 7 normal controls (34). Consistent with the key role of the IGF system in cancer progression, IGF1R and IGF2 levels were much higher in advanced stage (Stages III–IV) malignant tissue compared to early stages or endometrial hyperplasia. Hence, these studies suggest that over-expression of the IGF1R and IGF2 genes is associated with poor outcome in endometrial cancer.

## Interactions with Steroid Hormones

Evidence accumulated in recent years indicates that the biological activity of the IGF system is strongly associated with estrogen status (35). Estrogens increase IGF binding and IGF1R mRNA levels in breast cancer cells, suggesting that a potential mechanism by which estrogens stimulate breast tissue proliferation involves sensitization to the mitogenic effects of IGFs by enhancing IGF1R concentrations (36). In addition, estrogens modulate IGF signaling by regulating the expression of other members of the IGF family, including ligands, IGFBPs, and insulin receptor substrate-1 (IRS-1) (37).

While the interplay between the IGF and estrogen signaling pathways was initially dissected in breast cancer models, further studies investigated the impact of estrogens on IGF1 action in additional steroid hormone-dependent cancers, including endometrial tumors. Estradiol stimulates proliferation of the uterine epithelium via a mechanism that involves activation of the IGF1R (38). In turn, activation of PI3K/PKB leads to cyclin D1 nuclear accumulation and engagement with the cell cycle machinery. Nuclear accumulation of cyclin D1 results from the inhibition of glycogen synthase kinase 3β (GSK3β) activity, caused by inhibitory phosphorylation by PKB. Given that estradiol is the main risk factor for endometrial cancer, data are consistent with the notion that downstream activation of the IGF1-mediated pathway by mutation could play a major role in the progression to ER-independent tumors (38).

In addition to estrogens, the IGF1 pathway is tightly regulated by androgens. The interplay between IGF1R and the androgen receptor (AR) was mainly investigated in prostate cancer models. Contradictory results, however, were published regarding the pattern of IGF1R expression throughout the various stages of the disease. Thus, whereas a number of studies reported that the progression of the tumor from an androgen-dependent to an androgen-independent stage is associated with a major decrease in IGF1R expression levels, other studies showed sustained up-regulation of IGF1R (39, 40). The effect of wild type AR on IGF1R expression was examined by means of co-transfection assays using AR expression vectors, along with an IGF1R promoter construct. Results obtained revealed that wild type, but not mutant, AR increased IGF1R promoter activity (41). Finally, Pandini et al. (42) reported that androgens induced IGF1R up-regulation via a *non-genomic* AR pathway.

## Interactions with Growth Factors and Peptide Hormones

In addition to the interactions of the IGF pathway with steroid hormones, as described above, the endocrine and local activities of IGF1 are also modulated by complex, often bidirectional, interactions with other peptide hormones. A recent study investigated the association between IGF1R and vascular endothelial growth factor-C (VEGF-C) expression and lymphatic metastasis in 40 endometrial adenocarcinoma tumors and 14 normal endometrium samples (43). Immunohistochemical analyses revealed that IGF1R expression was associated with histological grade and lymph node metastasis, but not with surgical stage. Moreover, IGF1R and VEGF-C expressions were correlated in endometrial adenocarcinomas, and lymphatic vessel density was closely related to both. Abnormal IGF1R and VEGF-C expressions, therefore, might constitute important markers for lymph node metastasis of endometrial adenocarcinoma and might be potentially useful for evaluating disease prognosis.

Insulin-like growth factor-1 and epidermal growth factor (EGF) downregulation and fibroblast growth factor-2 (FGF2) up-regulation seem to constitute key features of endometrial cancer progression, as demonstrated in a collection of 30 cancer specimens that were compared to normal adjacent tissue. Pathologic disruption of mRNA co-expression patterns supports the notion of a cross talk between IGF1, EGF, and FGF2 signaling pathways in the promotion of endothelial cell proliferation and differentiation of endometrial cancer (44). Given that IGF1 ligand downregulation is usually correlated with IGF1R up-regulation, above studies are consistent with constitutive activation of the IGF1R signaling cascade in endometrial cancer.

## Regulation of IGF1R Gene Expression

As alluded above, the typical features of the IGF1R include: (i) potent anti-apoptotic and mitogenic capabilities (15), (ii) important roles in invasion, metastasis, and angiogenesis (17–19), and (iii) involvement in oncogenic transformation (15, 26). Evidence in support of a key role of IGF1R in malignancy is provided by the fact that IGF1R-null fibroblasts (derived from IGF1R KO embryos, a lethal condition) do not undergo transformation when exposed to cellular or viral oncogenes (15). The levels of expression of IGF1R as a determinant of IGF1 and IGF2 action and, in particular, the biological significance of IGF1R over-expression under pathological conditions, are still open questions. Nevertheless, IGF1R over-expression is regarded as a “*quasi*” universal pre-requisite for oncogenic transformation. This widely accepted viewpoint relies on the well-established concept that elevated IGF1R levels and enhanced IGF signaling are key events, indispensable for the cell to adopt proliferative/oncogenic pathways. Of importance, differences in IGF1R expression patterns exist between different cancer types. Thus, IGF1R over-expression is a typical feature of most pediatric cancers and other solid tumors (e.g., brain and kidney), whereas the situation with adult epithelial tumors (e.g., breast and prostate) is more complex and reduced IGF1R levels are often seen in advanced stage diseases (26).

## Regulation of IGF1R Expression by p53

**Figure 1:**
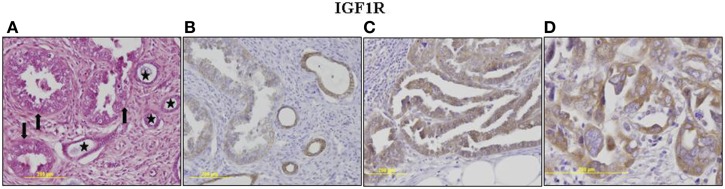
**Immunohistochemical staining of IGF1R in primary and metastatic USC**. **(A)** *Primary tumor*, neoplastic glands (arrows) adjacent to non-neoplastic endometrial glands (asterisks). H&E, ×200. **(B)** *Primary tumor*, weak cytoplasmic staining in <50% of cells (grade II) in neoplastic glands. Moderate staining in >50% of cells in adjacent non-neoplastic glands. **(C)** *Metastasis*, moderate cytoplasmic staining in nearly all neoplastic cells. **(D)** *Metastasis*, a focus of increased atypia. Moderate and strong staining in all neoplastic cells. Some of the immunohistochemical figures were shown in Ref. (22).

**Figure 2:**
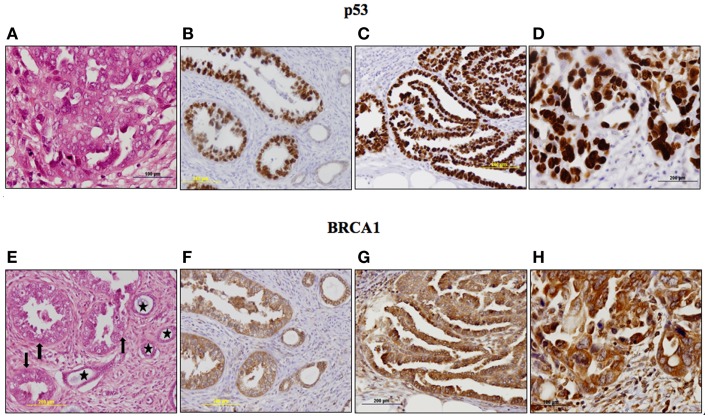
**Immunohistochemical staining of p53 (A–D) and BRCA1 (E–H) in primary and metastatic USC**. p53: **(A)** *Metastasis*, a focus of increased atypia in metastatic tumor. Enlarged, pleomorphic and bizarre nuclei, atypical mitoses. H&E, ×400. **(B)** *Primary tumor*, strong nuclear staining in nearly all neoplastic cells (grade IV). No staining in adjacent non-neoplastic glands. **(C)** *Metastasis*, strong nuclear staining in nearly all neoplastic cells (grade IV). **(D)** *Metastasis*, a focus of increased atypia. Very strong staining in all neoplastic cells (grade IV). BRCA1: **(E)** *Primary tumor*, neoplastic glands (arrows) adjacent to non-neoplastic endometrial glands (asterisks). H&E, ×200. **(F)** *Primary tumor*, moderate cytoplasmic staining in >50% of cells in neoplastic and non-neoplastic glands. **(G)** *Metastasis*, moderate and strong cytoplasmic staining in nearly all neoplastic cells. **(H)** *Metastasis*, a focus of increased atypia, strong staining in all neoplastic cells. Immunohistochemistry data were shown in Ref. (23).

The p53 pathway is activated in response to a wide variety of cellular stress signals. These insults include DNA damage and telomere shortening, hypoxia, spindle damage, heat and cold shock, inflammation, nitric oxide production, and activation of oncogenes by mutations. These stresses can potentially lead to a decrease in the fidelity of cell cycle progression and DNA replication, with an ensuing increase in mutation rates. The convergence of the p53 and IGF signaling pathways has been the focus of considerable basic and translational interest (45). In a recent study, we evaluated p53 and IGF1R expression in a group of 35 USC patients, of which 17 had metastatic tumors (22). Immunohistochemical analysis revealed that IGF1R was highly expressed in primary and metastatic USC tumors (Figure 1). Positive staining (grades II–IV) was observed in 94.3% of primary USC tumors and in all metastatic tumors. High IGF1R expression (grades III–IV) was recorded in 42.8% of the primary tumors and 77.8% of the metastatic tumors. p53 was expressed in 85.7% of primary tumors and 100% of metastases. The staining was strong (grade IV) in 68.6% of primary USC tumors and in all metastatic tumors (Figure 2).

**Figure 3:**
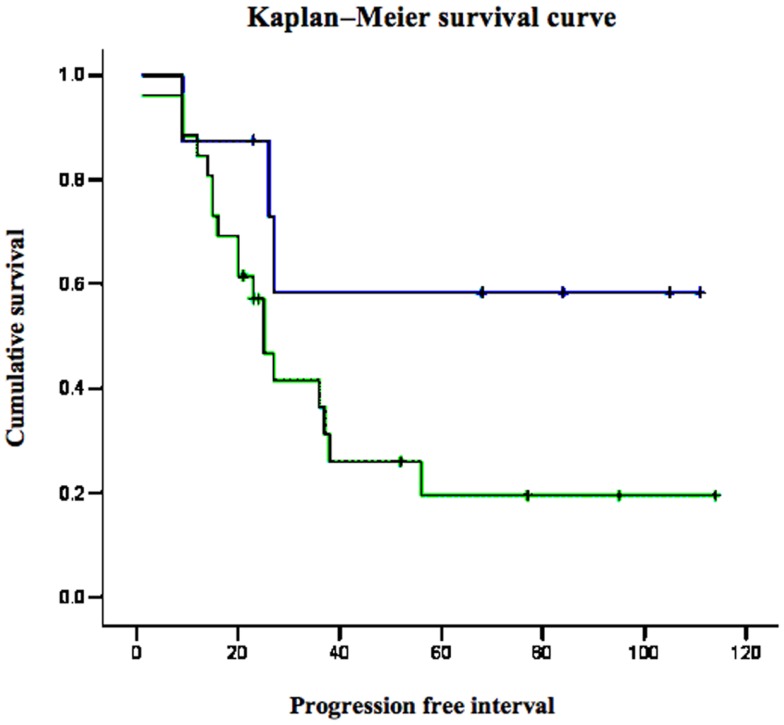
**Kaplan–Meier survival curve for p53-overexpressing and non-expressing tumors in women with USC**.

Survival analyses were conducted to evaluate the impact of p53 and IGF1R expression on endometrial disease prognosis. Data analysis revealed a negative correlation between p53 expression and survival. Specifically, 5-year survival was 12% in patients with strong p53 staining compared to 60% in patients with negative-to-moderate expression. Mean progression free interval was shorter in patients with strong p53 staining compared to patients with low p53 (28 vs. 58.2 months, respectively; *p* = 0.012) in univariate analysis. Kaplan–Meier survival analysis was performed for patients whose tumors exhibited over-expression of p53 and for patients whose tumors did not express p53 (Figure 3). Survival was significantly shorter in patients whose tumors over-expressed p53 compared to low p53 expressors (*p* = 0.009). No correlation between IGF1R expression and survival was observed.

**Figure 4:**
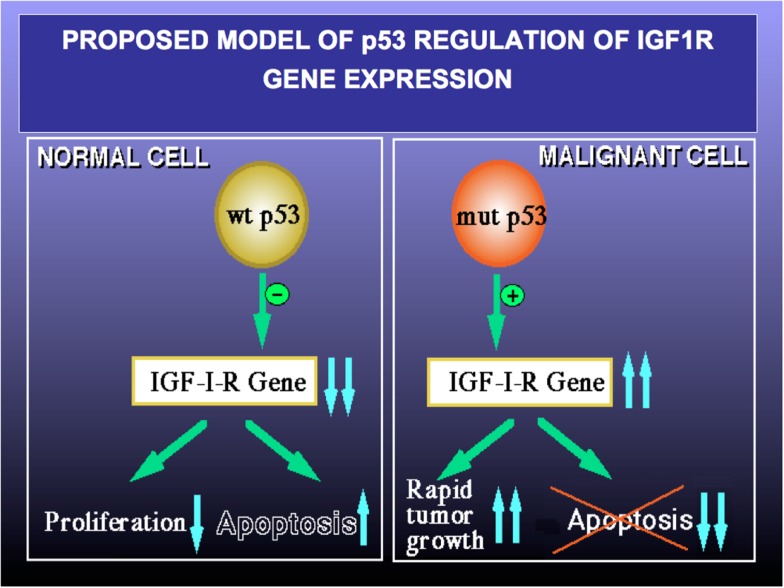
**Proposed model of p53 regulation of IGF1R gene expression**. Tumor suppressor p53 is a negative cell cycle regulator that prevents passage of damaged DNA to daughter cells. Wild type p53 suppresses IGF1R gene transcription, leading to reduced IGF1R levels and a decrease in IGF1- and IGF2-stimulated IGF1R phosphorylation. As a net result, cell proliferation is reduced and apoptosis is increased (left panel). *Loss-of-function* or *gain-of-function* mutations of p53 are common events in cancer (right panel). Mutant forms of p53 are able to transactivate the IGF1R gene, with ensuing increase in IGF1R biosynthesis. Augmented IGF1R expression and activation is a pre-requisite for tumor growth and is usually associated with abrogation of apoptosis (i.e., enhanced cell survival). A similar paradigm applies for BRCA1.

Finally, to examine the involvement of p53 in regulation of IGF1R gene expression in USC, the USPC-2 cell line (expressing a truncated mutant p53) was transiently co-transfected with a wild type or mutant p53 expression vector along with an IGF1R promoter-luciferase reporter plasmid. Results of luciferase measurements indicate that wild type p53 repressed IGF1R promoter activity by ~65%. Mutant p53, on the other hand, was unable to inhibit promoter activity. The mechanism of action of p53 was shown to involve interaction with zinc finger protein Sp1, a potent transactivator of the IGF1R gene. In summary, p53 regulates IGF1R gene expression in endometrial cancer via a mechanism that involves repression of the IGF1R promoter. The interplay between the p53 and IGF signaling pathways has major translational relevance. Pathologic deregulation of IGF1R gene expression as a result of tumor-specific, *loss-of-function* p53 mutations may lead to increased cell-surface IGF1R concentrations and enhanced IGF1R phosphorylation by locally produced and/or endocrine IGF1 and IGF2. As mentioned above, IGF1R activation constitutes a cardinal step in tumor progression (Figure 4). Elucidation of the transcription mechanisms and factors responsible for IGF1R gene expression will help to improve our ability to deliver IGF1R-directed therapies. These analyses, in addition, will be invaluable in predicting responsiveness to these therapies.

## Regulation of IGF1R Expression by BRCA1

Breast cancer susceptibility gene-1 is a tumor suppressor gene whose mutation has been associated with the appearance of breast and/or ovarian cancer at young ages. Breast cancer susceptibility gene-1 (BRCA1) participates in multiple biological pathways including DNA damage repair, transcriptional control, cell growth, and apoptosis (46). Similar to the paradigm described above for p53, BRCA1 has been shown to inhibit IGF1R transcription in breast, osteosarcoma, and ovarian cancer cell lines (47–49). These results suggest that a potential mechanism of action of BRCA1 involves suppression of IGF1R gene expression. In contrast, mutant BRCA1 proteins lack transcriptional activity and are impaired in their ability to suppress the IGF1R promoter, with resulting increases in IGF1R mRNA and IGF binding in mammary tumors. Consistent with the postulate that mutant BRCA1 may lead to deregulated IGF1R expression, a recent immunohistochemical analysis revealed significantly elevated IGF1R levels in primary breast tumors derived from BRCA1 mutation carriers, compared to sporadic tumors (50).

In the context of endometrial tumors, we investigated the rate of three predominant BRCA1/2 mutations in Jewish patients with USC and the relevance of carrier status to clinico-pathological features and survival (51). Overall, 8 of 31 patients (25.8%) included in the study were mutation carriers. Four were BRCA2 (6174delT) carriers and two each carried the BRCA1 (185delAG) and the BRCA1 (5382InsC) mutations. Given the high incidence of BRCA mutations in USC patients, it has been postulated that USC could be considered an integral component of the hereditary breast–ovarian cancer syndrome.

To investigate BRCA1 expression in USC and its correlation with IGF1R, immunohistochemical stainings were conducted in USC samples in paraffin blocks. The group comprised 35 patients with histologically confirmed USC, including 17 patients with metastases. Results revealed high BRCA1 expression in primary and metastatic USC (23). BRCA1 staining was mainly cytoplasmic. BRCA1 was expressed in all primary and metastatic tumors with strong staining in 71% (25/35) of primary tumors and 82.4% (14/17) of metastatic tumors. Metastatic tumors stained more intensely for BRCA1 (82.3% strong staining) compared to primary tumor sites (71.4% strong staining; *p* = 0.041; Figure 3).

To investigate the effect of BRCA1 on IGF1R promoter in endometrial cancer cells, co-transfection assays were performed using a BRCA1 expression vector along with an IGF1R promoter reporter (23). Results revealed that BRCA1 over-expression led to 46–65% decline in IGF1R promoter activity in two USC-derived cell lines. Measurement of endogenous IGF1R protein in USC cells corroborated the inhibitory role of BRCA1 on IGF1R expression and emphasized the physiological relevance of these results. Furthermore, BRCA1 over-expression led to a reduction in phospho-Akt, an important downstream mediator of IGF1R. In summary, these data emphasize the notion that tumor suppressor BRCA1 is involved in controlling the expression and action of the IGF axis in endometrial cancer. The convergence of IGF1R-mediated cell survival pathways and BRCA1-mediated tumor protective pathways is of major basic and clinical relevance.

## IGF1 Axis Targeting in Endometrial Cancer

IGF1 receptor targeting emerged in recent years as a very active area in cancer therapeutics. IGF1R targeting is expected to result in: (i) inhibition of IGF1R expression; (ii) blockade of ligand–receptor interaction; and/or (iii) impairment of receptor activation. Targeting methods are evaluated for their ability to: (i) inhibit cancer cell proliferation, survival, and anchorage-independent growth *in vitro*; (ii) reverse tumor growth and metastasis formation *in vivo*; and (iii) sensitize cancer cells to chemotherapy, radiotherapy, hormonal, and biological therapies. Various methodologies are currently being evaluated for their ability to down-regulate IGF1R expression and signaling. The most promising approaches at present are IGF1R monoclonal antibodies and IGF1R-selective low molecular weight tyrosine kinase inhibitors (TKIs). Humanized IGF1R antibodies are designed to prevent IGF1 binding, with ensuing receptor degradation, whereas TKI, on the other hand, are designed to inhibit IGF1R kinase activity without affecting receptor expression (52–60).

Several IGF1R antibodies were evaluated in Phase 1–3 clinical trials as monotherapy, as well as in combination with chemotherapy, radiotherapy, and/or additional antibodies. Currently, the greatest clinical impact of IGF system signaling inhibition for cancer treatment is prevention or reversal of resistance to anti-cancer therapies. Therapies directed against the IGF1R were shown to enhance the cytotoxic effects of conventional treatments. In a Phase 1 study of AMG-479 (Ganitumab, Amgen, a monoclonal IGF1R antibody) that enrolled 33 epithelial ovarian cancer patients, 3 had an objective response and 5, stable disease (NCT00718523). The dose-limiting toxicity of this agent was thrombocytopenia; additional adverse effects included arthralgia, diarrhea, and hyperglycemia (52). Two Phase 1 trials evaluated the activity of CP-751, 871 (figitumumab, Pfizer), an IGF1R monoclonal antibody, in combination with docetaxel or carboplatin and paclitaxel in patients with advanced solid tumors and reported that this drug combination was well tolerated (53). Phase 1 and 2 trials in small cell lung cancer, colorectal, pancreatic, and ovarian cancers and in other solid tumors are currently underway. However, few of these trials have progressed to or completed Phase 3 studies. Two Phase 3 trials testing figitumumab in conjunction with chemotherapy or combined with EGF receptor inhibitor (erlotinib) in advanced and in relapsed non-small cell lung cancer, were discontinued after data showed significant side effects (54).

Although most of these antibodies do not bind to the insulin receptor (INSR), some of them partially cross-react with the INSR leading to hyperglycemia in clinical studies. The potential effect of IGF1R antibodies on INSR signaling is of special concern, given that these antibodies can co-target or alter INSR function, leading to insulin resistance and adverse effects on glucose and carbohydrate metabolism. On the other hand, INSR targeting could be potentially advantageous, because specific inhibition of the INSR in the tumor might increase the effective anti-tumoral activity (27, 28, 55). Preliminary results from Phase 1 trials in patients with advanced cancer treated with CP-751, 871 or IM Clone’s A12 antibodies showed only infrequent, mild, transient hyperglycemia, with no dose-limiting toxicity.

In addition to IGF1R antibodies, a series of low molecular weight TKIs have demonstrated tumor growth inhibitory properties in experimental models. In general, these therapies indiscriminately inhibit both IGF1R and INSR kinase domains, as these enzymatic domains are closely related (56). However, several TKI are more selective to the IGF1R and have a 15- to 30-fold increased potency for IGF1R kinase inhibition compared to INSR kinase inhibition (57). NVP-AEW541 (Novartis AG, Basel, Switzerland), an orally available, IGF1R-specific TKI, inhibited IGF1R signaling in tumor xenografts and significantly reduced the growth of IGF1R-driven sarcomas (57). OSI-906 (Astellas Pharma Inc., Tokyo, Japan) is a potent, selective, orally bioavailable, dual IGF1R/INSR TKI, which has demonstrated *in vivo* efficacy in tumor models and is currently in clinical evaluation.

Several *in vivo* studies demonstrated that inhibition of the IGF1 and IGF2 ligands using neutralizing antibodies resulted in potent antitumor activity and offer an effective approach to selectively target both the IGF1R and INSR-A signaling pathways (58). However, only MEDI-573, a fully human antibody that neutralizes IGF1 and IGF2 and inhibits IGF signaling through both pathways, progressed into clinical studies (advanced solid tumors and metastatic breast cancer). To the best of our knowledge, this targeted strategy has never been evaluated in gynecologic malignancies.

As mentioned above, there is a tight interaction between the insulin and IGF1 pathways (59, 60). In fact, both INSR and IGF1R share most of their downstream cytoplasmic mediators. Metformin (*N*,*N*-dimethylbiguanide), an oral anti-hyperglycemic agent of the biguanides family, is undergoing a renaissance because of its potential as a cancer therapy, along with its traditional role in treating diabetes. Recent studies reported that metformin use was associated with a significant decrease in the incidence of cancer (61). *In vitro* studies suggested that metformin inhibits cancer cell growth by activating adenosine monophosphate protein kinase (Ampk), by inactivating the mammalian target of rapamycin (mTOR), and by decreasing the activity of the mTOR effector S6K1 (62). In the specific context of endometrial cancer, studies demonstrated a significant antiproliferative activity of metformin in Type I endometrial cancer. Cantrell et al. (63) reported that metformin treatment resulted in G1 arrest, apoptosis and decreased cell proliferation in ECC and Ishikawa endometrial cells. This effect was partially mediated through Ampk activation and subsequent inhibition of the mTOR pathway. Furthermore, metformin was shown to enhance the sensitivity of type I endometrial cells to cisplatin and taxol chemotherapy (64, 65).

In a recent study, we explored the effects of metformin in USC cells. Our data provided evidence that metformin down-regulated the expression of both IGF1R and INSR. In addition, metformin induced apoptosis and inhibited proliferation and migration of USC cell lines including both wild type and mutant p53. These data suggest that metformin therapy could be a novel and attractive therapeutic approach for endometrial cancer, in general, and USC, in particular (66). Of interest, a recent study demonstrated that metformin inhibits IGF1R up-regulation in prostate cancer cells *via* a mechanism that involves disruption of membrane-initiated androgen signaling (67).

Finally, vorinostat, also known as suberanilohydroxamic acid (SAHA), is a novel histone deacetylase inhibitor, representing a new class of potential antitumor agents (68). Vorinostat induced growth arrest, differentiation, and apoptosis in a variety of transformed cells, including prostate, leukemia, breast, and colon cancers. Vorinostat has undergone evaluation in a number of clinical trials (69). In endometrial cancer, the combination of vorinostat and caspase-8 inhibitor led to high anti-tumoral activity (70). In a recent study, we evaluated the antiproliferative activity of vorinostat in Type I and II endometrial cancer cells (71). Vorinostat treatment induced apoptosis in both cell types, abolished the anti-apoptotic activity of IGF1, and led to a significant decrease in colony forming capability.

## Identification of Biomarkers

As mentioned above, the IGF axis has gained interest as a risk factor with a potential role in the progression of endometrial carcinoma. Several studies showed a significant correlation between components of the IGF system and endometrial cancer risk (20, 29, 59). Nevertheless, the response to therapy targeting IGF1R was relatively low due to the lack of predictive tumor biomarkers that could assist in selecting patients who would benefit from the proposed treatment. Growing clinical evidence suggests a potential correlation between biomarkers related to the IGF1R pathway and clinical benefits from IGF1R-targeted therapies. High IGF1R expression and elevated circulating IGF1 levels were shown to be correlated with improved response to IGF1R-targeted therapies (72, 73). In addition, increased IGF1R nuclear localization was associated with better overall survival in sarcoma patients treated with IGF1R antibody (74). In a recent review, Michael Pollak suggested that IGF1R levels and the presence of IGF1R autocrine loops are possible predictive biomarkers for IGF1R-targeted therapy (60). It was also suggested that the presence of activating mutations downstream of IGF1R would induce resistance to IGF1R-targeted therapy.

As discussed above, our studies provided evidence for functional and physical interactions between the IGF signaling pathways and tumor suppressors p53 and BRCA1. We demonstrated that IGF1R gene transcription rate is dependent on a number of stimulatory nuclear proteins and is also modulated by negative transcriptional regulators, including p53/p63/p73 (45, 75) and BRCA1 (46–50). The level of expression of the IGF1R gene is ultimately determined by complex interactions between stimulatory and inhibitory transcription factors. Aberrant deregulation of these regulatory loops can lead to enhanced IGF1R expression, a pre-requisite for malignant transformation. Furthermore, it was demonstrated that the mutational status of p53 and, probably, BRCA1 has a major impact on response to IGF1R-targeted therapy (22, 23). Finally, the potential role of p53 and BRCA1/2 as biomarkers for IGF1R-directed therapies in endometrial cancer (and most likely other types of cancer) must be confirmed by larger cell-based and patient analyses.

## Conclusion

It is well-established that the IGF hormonal network plays an important role in normal cell growth and differentiation as well as in the establishment and maintenance of a malignant phenotype. Changes in IGF1 expression and signaling play key roles in the regulation of normal uterine physiology. In addition, there is a causative linkage between deregulated expression and activation of IGF axis components and endometrial cancer. As a corollary, targeted IGF1R therapy has emerged as a biologically plausible approach. The physical and functional interactions between circulating and locally produced IGF components and steroid hormones and their receptors are of major importance under both physiological and pathological conditions. Likewise, control of IGF1R expression and action by tumor suppressors (e.g., p53, BRCA1) involved in the etiology of gynecological cancer has a major impact on cell’s fate and, consequently, is of high translational relevance. Further basic and clinical research is needed to achieve better therapeutic outcomes in endometrial cancer.

## Conflict of Interest Statement

The authors declare that the research was conducted in the absence of any commercial or financial relationships that could be construed as a potential conflict of interest.

## Abbreviations

AR, androgen receptor; BMI, body mass index; BRCA1, breast cancer susceptibility gene-1; ER, estrogen receptor; IGF1, insulin-like growth factor-1; IGF1R, IGF1 receptor; IGFBP, IGF-binding protein; INSR, insulin receptor; IRS-1, insulin receptor substrate-1; PR, progesterone receptor; TKI, tyrosine kinase inhibitor; USC, uterine serous carcinoma.
